# The Open State Selectivity of the Bean Seed VDAC Depends on Stigmasterol and Ion Concentration

**DOI:** 10.3390/ijms22063034

**Published:** 2021-03-16

**Authors:** Hayet Saidani, Marc Léonetti, Hanna Kmita, Fabrice Homblé

**Affiliations:** 1 Structure et Fonction des Membranes Biologiques, Université Libre de Bruxelles (ULB), Boulevard du Triomphe CP 206/2, B-1050 Bruxelles, Belgium; hayettesaiidany@gmail.com; 2 Laboratory of Functional Neurophysiology and Pathology, Research Unit, UR/11ES09, Department of Biological Sciences, Faculty of Science of Tunis, University Tunis El Manar, 1068 Tunis, Tunisia; 3 Université de. Grenoble Alpes, CNRS, LRP, 38000 Grenoble, France; leonettm@univ-grenoble-alpes.fr; 4 Department of Bioenergetics, Institute of Molecular Biology and Biotechnology, Faculty of Biology, Adam Mickiewicz University, Uniwersytetu Poznańskiego 6, 61-614 Poznań, Poland; kmita@amu.edu.pl

**Keywords:** VDAC, selectivity, mitochondria, protein-lipid interaction, stigmasterol, phaseolus, plant seeds, plant biochemistry

## Abstract

The voltage-dependent anion channel (VDAC) is the major pathway for metabolites and ions transport through the mitochondrial outer membrane. It can regulate the flow of solutes by switching to a low conductance state correlated with a selectivity reversal, or by a selectivity inversion of its open state. The later one was observed in non-plant VDACs and is poorly characterized. We aim at investigating the selectivity inversion of the open state using plant VDAC purified from *Phaseolus coccineus* (PcVDAC) to evaluate its physiological role. Our main findings are: (1) The VDAC selectivity inversion of the open state occurs in PcVDAC, (2) Ion concentration and stigmasterol affect the occurrence of the open state selectivity inversion and stigmasterol appears to interact directly with PcVDAC. Interestingly, electrophysiological data concerning the selectivity inversion of the PcVDAC open state suggests that the phenomenon probably does not have a significant physiological effect in vivo.

## 1. Introduction

The exchange of inorganic ions and metabolites between the mitochondria and the cytosol is essential for numerous mitochondrial functions. In plants, as in other eukaryotic organisms, a wide variety of transporters (ion channels, carriers and ABC transporters) mediate a selective transport through the mitochondrial inner membrane (MIM) [1,2,3,4,5]. In contrast, the voltage-dependent anion channel (VDAC) is the major transport pathway in the mitochondrial outer membrane (MOM) for compounds as diverse as inorganic ions (e.g., K^+^, Na^+^ and Cl^−^), metabolites (e.g., ATP and AMP) and large macromolecules such as tRNA [6].

Several clues suggest that VDACs are involved in the plant response to abiotic and biotic stresses [6,7,8,9,10]. The modification of the membrane lipid composition is a hallmark of plant stress responses and its effect on mitochondria function has long been recognized [11,12,13]. Phosphatydylcholine and phosphatydyletanolamine are the most abundant constituents of the MOM lipid bilayer [14] and make up to ca. 80–90% of the total amount of MOM phospholipids in plants [15,16,17,18]. The gating of the major VDAC isoform isolated from *Phaseolus coccineus* (PcVDAC) is sensitive to the surrounding phospholipids [19]. It has been shown that cholesterol and phytosterols (stigmasterol and sitosterol) modulates differentially the PcVDAC selectivity, and stigmasterol is most effective to increase the selectivity of the open state. Moreover, only stigmasterol alters the voltage-dependence of the PcVDAC [20]. This indicates that lipid-protein interactions are important for the proper functioning of PcVDAC.

High-resolution structures of mammalian and insect VDACs reveal a beta-barrel structure consisting in 19 anti-parallel β-strands with an N-terminal helix folded into the pore [21,22,23]. It has a wide inner pore diameter ranging between 3 and 1.5 nm. Although the high-resolution structural data are lacking for plant VDACs, several data suggest that plant VDACs share the same fold. It has been shown that: (1) the pore size determined for potato tuber VDAC by AFM is consistent with the backbone–backbone diameter of the mammalian 3D structures [24]; (2) spectrophotometric (CD, FTIR) and bioinformatics studies indicate that the secondary structure content of PcVDAC is close to that of the atomic resolution structures of the mammalian VDAC1 [6,25,26] and (3) the VDAC supramolecular organization is the same in plants and in other organisms [24,27]. In addition, 3D model of PcVDAC was shown to display features such as electrostatic energy ion profile, β-strand dynamics, distribution of the hydrophobic and charged residues as well as ion permeation akin to those of the mouse VDAC1 crystal structure [6,28].

VDAC from all organisms have similar basic electrophysiological properties (conductance, selectivity and voltage-dependence) [6,29]. At low voltages (|V| < 20 mV, where |V| is the modulus of V), VDAC is in the open state and is more permeable to anions, while at higher applied voltages (|V| > 20 mV), VDAC switches to lower conducting substates featuring a cation selectivity [30]. Since most metabolites transported through VDAC have a net negative charge, the change in VDAC conductance at higher voltages might thus regulate their flux through MOM [31]. Accordingly, ATP permeates VDAC in the open state but not in the low conductance substates [32,33]. A new paradigm has emerged from these findings, namely the voltage gating involves a tight coupling between the change in conductance and VDAC selectivity, thus allowing the regulation of the ATP flow through MOM [34,35].

There are, however, experimental data that challenges the conductance-selectivity coupling. In *Neurospora crassa*, the VDAC selectivity of the low conductance substates can be either cation- or anion-selective [36]. In *Dictyostellium discoideum*, the VDAC open state was either anion- or cation-selective depending on the ionic strength of the solution [37]. Moreover, in both *N. crassa* and mammalian VDACs, the open state can randomly switch between anion and cation selectivity without change in conductance [38]. Altogether, the data suggests that in some conditions, yet undetermined, the conductance and selectivity are not coupled. The molecular determinants of the selectivity inversion of the VDAC open state are unknown. Since VDAC presumably regulates the transport of metabolites involved in the oxidative phosphorylation [39,40,41], it has been hypothesized that the inversion of the open state selectivity might be an alternative way to regulate their flow through MOM and thereby the cell physiology [42].

The deciphering of the mechanism underpinning the regulation of the VDAC selectivity is essential as most metabolites exchanged between mitochondria and the cytosol are anions indispensable for the mitochondrial respiration. As VDACs from all organisms share similar functional and structural properties, we might anticipate that plant VDACs would also display an open state selectivity inversion. Here, we address this issue by studying: (1) the effect of ion concentration and the stigmasterol presence on the selectivity of the PcVDAC open state using planar lipid bilayer (PLB) electrophysiology and (2) the effect of the stigmasterol presence on the PcVDAC structure dynamics by measuring the VDAC proton/deuterium exchange kinetics by means of attenuated total reflection Fourier transform infrared (ATR-FTIR) spectroscopy. Based on our data, we discuss the physiological relevance of the cationic open state for the regulation of metabolite transport through the MOM as well as the role of stigmasterol in the PcVDAC selectivity.

## 2. Results

### 2.1. Selectivity Inversion

**Figure 1:**
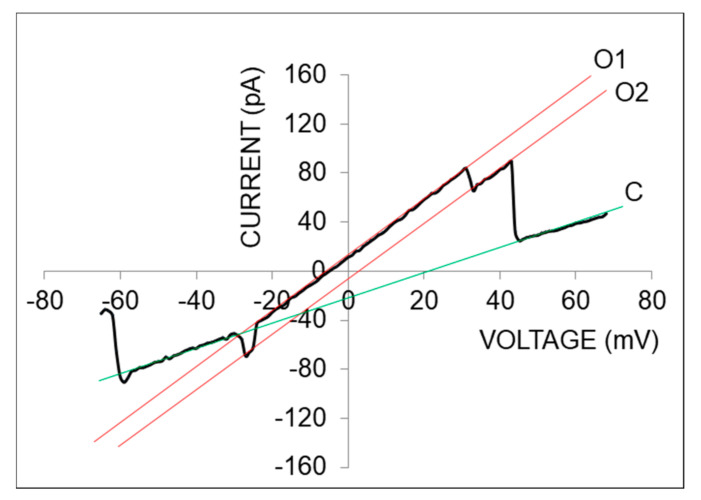
Typical current-voltage curve of single PcVDAC (*Phaseolus coccineus* voltage-dependent anion channel) recorded in the presence of a ten-fold KCl concentration ratio ((1/0.1 M KCl; Trans/Cis) across the membrane (12 replicates). The solutions were buffered at pH 7.5 with 10 mM HEPES-KOH. The straight lines drawn through the graph correspond to either the canonical anion open state (O1) or the more cation selective open state (O2) or the cation selective lower-conductance substate (C). A positive reversal potential (V (I = 0)) indicates a higher selectivity towards cations whereas a negative reversal potential indicates a higher selectivity towards anions.

The reversal potential (E_rev_) viz., the voltage at which there is no net ion flux through the channel (I = 0) in the presence of a salt concentration gradient across a channel, is commonly used to evaluate the channel selectivity. To observe the PcVDAC selectivity inversion, a single channel was reconstituted in a planar lipid bilayer made of soy lipids in the presence of 1 M KCl on both Cis and Trans side of the membrane. Then, ten-fold (1/0.1 M) KCl gradient was formed by decreasing the KCl concentration in the Cis compartment, and the current flowing through the PcVDAC in response to a linear voltage ramp was recorded. In these experimental conditions ([KCl]_Trans_ > [KCl]_Cis_), a negative (positive) E_rev_ value indicates an anion (cation) selectivity, respectively. The representative current-voltage curve recorded in the presence of the KCl gradient indicated the presence of two different selectivities (O1 and O2) for the open state and one lower-conductance substate (C) (Figure 1). The open state was slightly selective to either anions (E_rev_ = −5 mV) or cations whereas the substate was cation selective (+20 mV). The slope of the current-voltage curve is a measure of the channel conductance. The two open states O1 and O2 had a similar conductance (2.2 nS and 2.3 nS, respectively) whereas the conductance of the lower-conductance substate was distinctly lower, i.e., 0.6 nS.

### 2.2. Effect of Concentration Gradient and Stigmasterol on the Selectivity and the Conductance

#### 2.2.1. Multi-Channel Experiments

The effect of the KCl gradient on the selectivity was studied using multi-channel lipid bilayer. In all cases, the concentration was fixed in the Trans compartment and the Cis compartment was perfused with a low concentration to generate a given concentration ratio (2, 3, 4, 6, 10, 20 and 30) between the two compartments. To investigate the ion concentration effect, these experiments were performed using two different fixed concentrations in the Trans compartment (1.0 and 0.1 M KCl). For historical reasons, the electrophysiological studies on VDAC were mostly carried out in a non-physiological 1 M salt solution. However, it has been shown that the functional properties of PcVDAC is significantly affected by the ion concentration [19,20,28] and thus, 0.1 M was chosen as an approximate of the ionic strength for plant cells.

**Figure 2:**
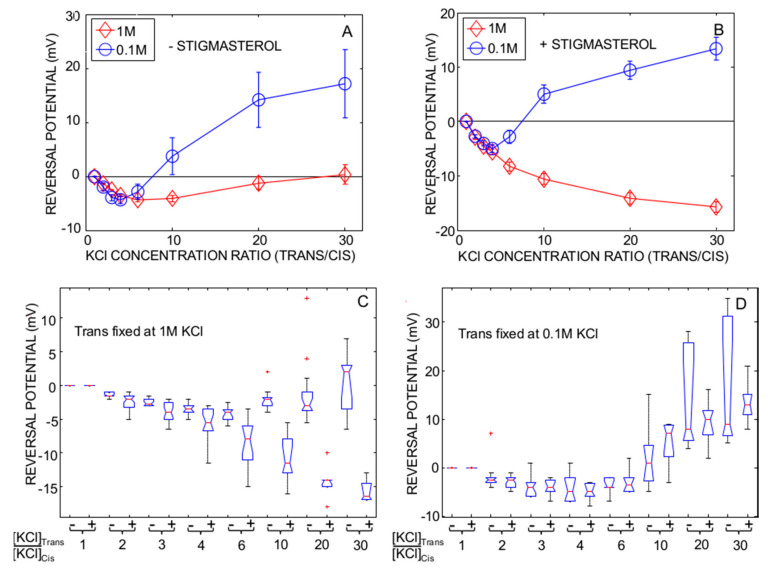
Changes in the reversal potential with KCl concentration ratio (Trans/Cis). (**A**,**B**), the ion concentration in the Trans compartment was fixed at either 1 M (red diamond) or 0.1 M (blue circle). To generate a given concentration ratio (2, 3, 4, 6, 10, 20 and 30) between the Trans and Cis compartments, the latter was perfused with low concentration KCl solutions. The solutions were buffered at pH 7.5 with 10 mM HEPES-KOH. The experiments were performed in the absence ((**A**), number of replicates: 7–19) or in the presence of 10% stigmasterol ((**B**), number of replicates: 5–28) in the membrane. (**C**,**D**), one-way ANOVA for the effect of stigmasterol with either high KCl concentration (**C**) or low KCl concentration (**D**) in the Trans compartment. The reversal potentials was measured in the absence (**-**) or in the presence (**+**) of stigmasterol at different KCl concentration ratio. (*p* < 0.001). A red cross indicates outliers.

In the absence of stigmasterol, the dependence of the reversal potential on the KCl gradient displayed a minimum at KCl concentration ratio of 4 (Figure 2A). Below this ratio value, E_rev_ was negative indicating that PcVDACs were in the open anionic state (O1). Above the concentration ratio value of 4, the E_rev_ increased towards positive values indicating that the occurrence of the more cationic O2 state increased. Note that all E_rev_ data were lower than the threshold voltage for triggering the lower-conductance substate (C). Therefore, in these multi-channel experiments, the measured E_rev_ is a weighted average of the reversal potentials of the individual channels, which might be in either O1 or O2 state.

In the presence of stigmasterol (Figure 2B), the dependence of the reversal potential on the concentration ratio was akin to that observed in the absence of stigmasterol in the presence of low KCl concentration in the Trans compartment (0.1 M) but not in the presence of high KCl concentration (1 M) in the Trans compartment. In the latter case, PcVDAC was in the more anionic O1 state at all tested concentration gradients. These results indicated that the PcVDAC selectivity was affected by the ion strength of the solution. To visualize statistical analysis of data shown in Figure 2A,B, we plotted the notch box for each KCl concentration ratio in the presence and in the absence of stigmasterol (Figure 2C,D). This one-way variance analysis featured a low *p*-value (*p* < 001), which indicates that at least one group is different from the others. The results indicated that there was a statistically significant effect of stigmasterol in the presence of fixed 1 M KCl in the Trans compartment at concentration ratios larger than 4 but not at low ionic strength in the presence of 0.1 M KCl in the Trans compartment. This conclusion was supported by a multiple pairwise comparison test performed to determine which group means are different from others (Figure S1: Multiple pairwise comparison).

#### 2.2.2. Single-Channel Experiments

**Figure 3:**
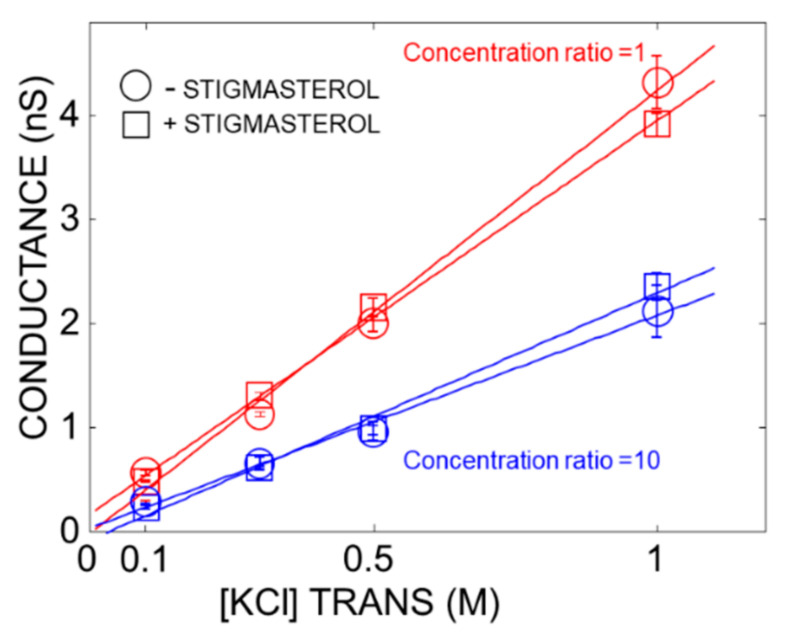
Effect of ion concentration on the PcVDAC conductance. The experiments were performed either in the presence of identical ion concentration on each side of the membrane (red symbol) or in the presence of a concentration ratio [KCl]_Trans_/[KCl]_Cis_ = 10 (blue symbol). The membrane was either free of stigmasterol (circle symbol, number of replicates: 3–19) or doped with 10% stigmasterol (square symbol, number of replicates: 5–9). The goodness of the linear fits are r^2^ > 99 (red circle), r^2^ > 99 (red square), r^2^ > 99 (blue circle) and r^2^ > 99 (blue square). The solutions were buffered at pH 7.5 with 10 mM HEPES-KOH.

We performed single-channel experiments to get a deeper understanding of the selectivity of the PcVDAC open state. The selectivity was measured as for multi-channel experiments. To evaluate the single-channel conductance, 10 mV voltage pulse was applied across the lipid bilayer and the resulting single-channel current was recorded (Figure S2: Channel conductance). The relationship between the single-channel conductance and the ion concentration is shown in Figure 3. Under the control symmetric conditions ([KCl]_Trans_ = [KCl]Cis; concentration ratio = 1), the magnitude of the conductance was typical for the single-channel O1 open state of the PcVDAC.

In agreement with the available data, there was a linear concentration-conductance relationship. This indicated that PcVDAC stayed in the open state at all the concentrations tested. The linear dependence was also observed in asymmetric conditions ([KCl]_Trans_ > [KCl]_Cis_) in the presence of a constant gradient (concentration ratio = 10). However, the magnitude of the conductance was lower than that of the control because there was ten times lower KCl concentration in the Cis compartment. The data shown in Figure 3 indicated that stigmasterol had no significant effect on the concentration-conductance correlation (see also Figure S2: Channel conductance).

**Figure 4:**
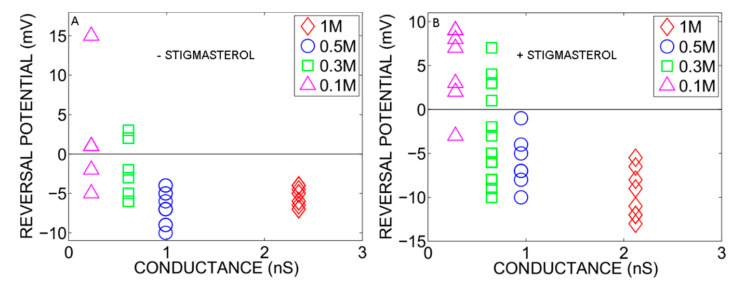
Effect of the ionic strength on the single-channel selectivity. The reversal potential was measured in the presence of constant ion concentration ratio of [KCl]_Trans_/[KCl]_Cis_ = 10. The ion concentration in the Trans compartment is shown in the insert. The reversal potential of each single-PcVDAC studied is plotted as a function of the average single-channel conductance determined for each ionic strength. The membrane was either free of stigmasterol (**A**) or doped with 10% stigmasterol (**B**). The solutions were buffered at pH 7.5 with 10 mM HEPES-KOH.

The effect of the ion concentration on the single-channel selectivity was measured in the presence of concentration ratio of 10 ([KCl]_Trans_ > [KCl]_Cis_) (Figure 4). The E_rev_ of single-channels was plotted as a function of the average single-channel conductance corresponding to the ion concentration in the Trans compartment (see Figure 3). The data collected in the presence of fixed concentration gradient showed that the probability to invert the selectivity increased at low ion concentrations. This is consistent with the data shown in Figure 2 at concentration ratio of 10. Independently of the ionic strength of the experimental solution, stigmasterol did not affect the PcVDAC conductance suggesting that PcVDAC remains in the open state in all the experimental conditions tested.

**Figure 5:**
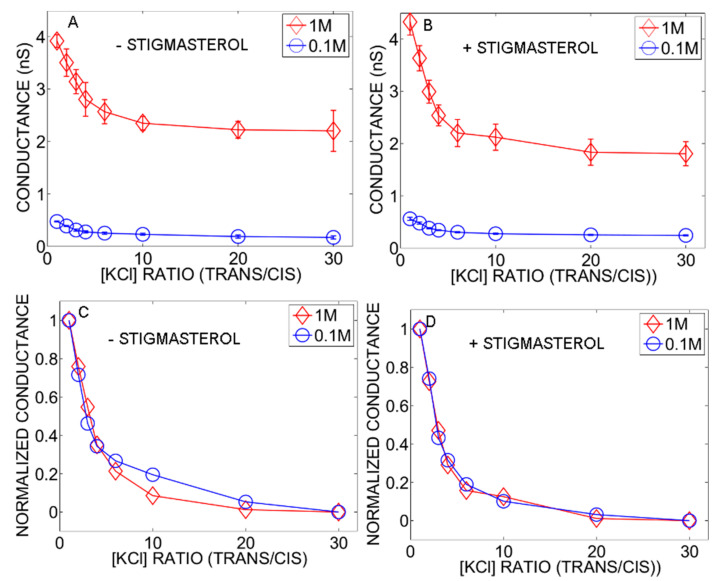
Changes in single-channel conductance with KCl concentration ratio (Trans/Cis). The ion concentration in the Trans compartment was fixed at either 1 M (red diamond,) or 0.1 M (blue circle). The solutions were buffered at pH 7.5 with 10 mM HEPES-KOH. The experiments were performed in the absence ((**A**,**C**), number of replicates: 4–9) or in the presence of 10% stigmasterol ((**B**,**D**), number of replicates: 3–7) in the membrane. The data shown in A and B were normalized to span between 0 and 1 which is shown in C and D, respectively.

The single-channel conductance was also estimated at different ion concentration ratios using fixed ion concentration (1 M or 0.1 M) in the Trans compartment. This protocol was the same as that used for multi-channel experiments (see Section 2.2.1). The experiments were done in the presence or in the absence of stigmasterol (Figure 5). At both high and low ion concentrations (1 M or 0.1 M), the single-channel conductance decreased monotonically as the concentration ratio increased and the magnitude of the single-channel conductance was much lower with fixed 0.1 M KCl concentration in the Trans compartment (Figure 5A,B). For easier comparison, the data recorded in 1 M or 0.1 M KCl were normalized so that the single-channel conductance spanned between 0 and 1 (Figure 5C,D). The data at high concentration superimposed to that at low concentration. This is strong evidence indicating that the single-channel conductance reflected the open state at all concentration ratios. In agreement with the results shown in Figure 2, stigmasterol had no effect on the single channel conductance.

### 2.3. Deuteration Kinetics

The amide groups of the protein backbone play a central role in determination of the protein structure and function, and can be studied by infrared spectroscopy. Notably, replacement of amide hydrogen by deuterium is extremely sensitive to environment and the kinetics of exchange allows the detection of structural and dynamics changes in membrane proteins surrounded by their lipid environment.

**Figure 6:**
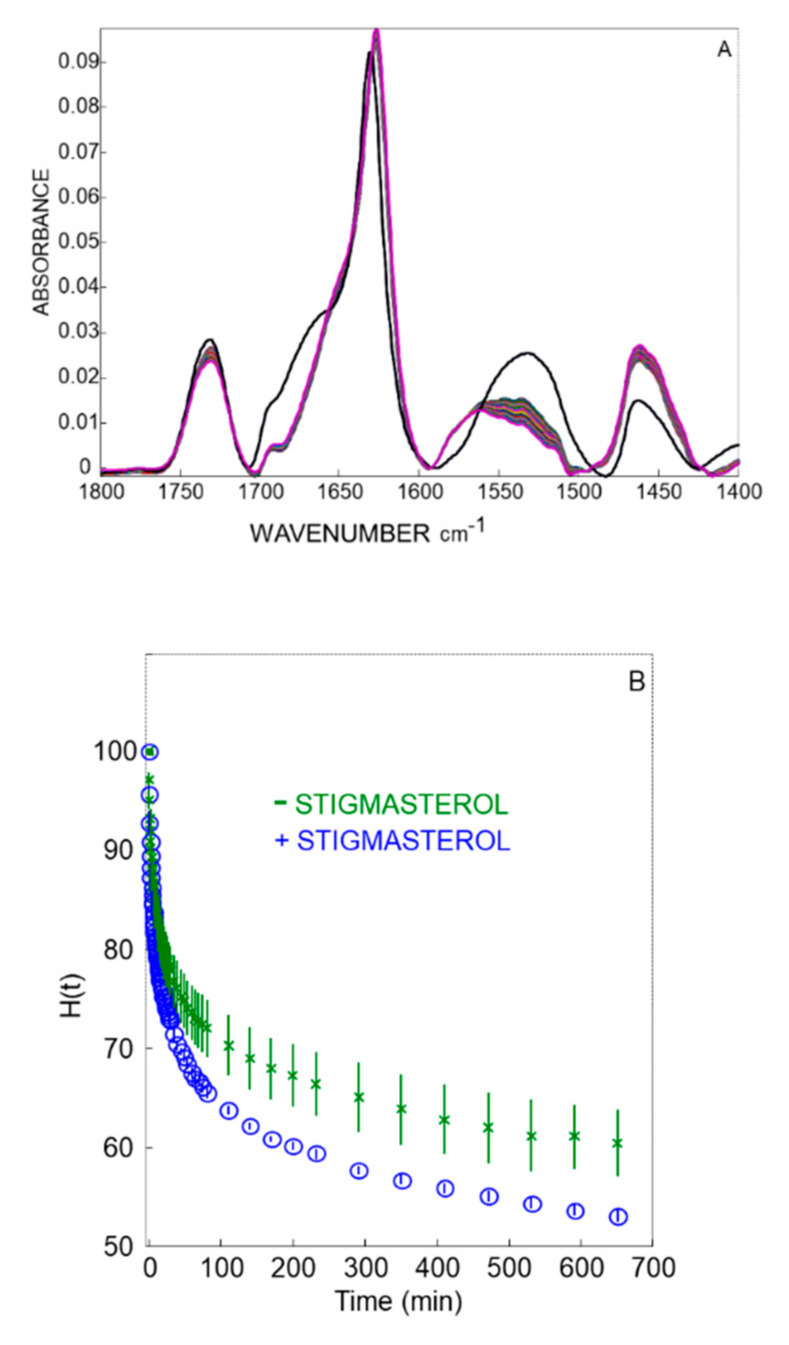
Hydrogen/deuterium exchange kinetics of PcVDAC reconstituted in liposomes. (**A**), superposition of the ATR-FTIR spectra in the course of the deuteration. The black spectrum corresponds to the undeuterated PcVDAC. The amide I band was integrated between 1700 and 1600 cm^−1^. The amide II band was integrated between 1590 and 1505 cm^−1^ and decreased upon deuteration. (**B**), Hydrogen/deuterium exchange kinetics reported as percent non-exchanged hydrogen. Deuteration percentage was evaluated from the evolution of the amide II/amide I area ratio upon exposure to D_2_O. The experiments were performed in the absence (number of replicates: 6) or in the presence of 10% stigmasterol (number of replicates: 3).

The deuteration kinetics was recorded during 11 h and the infrared spectra were plotted against the wavenumber (Figure 6A). The black spectrum is the non-deuterated spectrum recorded just before the deuteration kinetics. The band peaking at ~1730 cm^−1^ is assigned to the C=O stretching motion of membrane phospholipids. The Amide I vibration (1700–1600 cm^−1^) arises mainly (70−80% of the potential energy) from the C=O stretching motion of the amide and is sensitive to the backbone secondary structure. It is therefore commonly used for the identification of the secondary structure components and their relative proportion in the protein. Notably, the presence of peaks at 1626 cm^−1^ and 1692 cm^−1^ is a salient feature of β-barrel channel proteins [6,25]. The Amide II vibration (1590 and 1505 cm^−1^) is indicative of the amide N-H bending motion (40−60% of the potential energy) and is used to monitor the degree of proton/deuterium exchange. Upon deuteration, the Amide II vibration shifts to 1490−1425 cm^−1^, mixes with other vibrations (lipid methylene scissoring motion) and forms a new band named amide II’ whose intensity increases with deuteration [43,44].

To minimize variations of the overall spectral intensities related to film swelling upon hydration in the first minutes of the measurement, the amide II/amide I area ratio was determined for each spectrum. Thus, the ratio of amide II/amide I area was preferred to display the evolution of the hydrogen/deuterium exchange kinetics, and expressed as percent of non-exchanged amide protons (H(t)). The non-deuterated spectra were taken as H(t) = 100%, whereas the H(t) = 0% value corresponds to a zero absorption in the amide II region (full deuteration). The time course of the proton/deuterium reaction is shown in Figure 6B. In the absence of stigmasterol, the decrease in the intensity of the amide II band reflected ∼40% of the protein backbone amide protons undergoing hydrogen/deuterium exchange. The remaining absorption in D_2_O represented peptide groups that were not exchanged after 11 h of deuteration. In the presence of stigmasterol, 10% increase in hydrogen/deuterium exchange occurred during the first 3 h of the kinetics. Then, the time course of hydrogen/deuterium exchange was parallel to that recorded in the absence of stigmasterol.

## 3. Discussion

The regulation of the VDAC selectivity is an essential functional process for the control of metabolite flow through MOM, notably for ATP. The anion selectivity of the VDAC open state has long been recognized [35,45,46]. It is commonly stated that VDAC is a gating channel that upon switching to a low conductance undergoes selectivity reversal (i.e., becomes more permeable to cations) resulting in inhibition of the transport of anionic metabolites [32,33,41]. The mechanism underpinning the change in conductance and its coupling to the change in selectivity is still a matter of discussion.

An alternative mechanism of selectivity regulation consisting in a selectivity inversion of the open state between more anionic (O1) and more cationic (O2) was observed in fungi and mammal VDACs [38]. We showed that this O1⬄O2 transition also occurred in the *Phaseolus coccineus* PcVDAC reconstituted in PLB system. This indicates that the selectivity inversion is a common feature of reconstituted VDACs of different eukaryotes. Moreover, our data indicates that the O1⬄O2 transition is affected by the ion concentration, the magnitude of the concentration ratio across the membrane and the presence of stigmasterol in the membrane.

### 3.1. Selectivity Inversion in the Absence of Stigmasterol

Searching for the selectivity inversion of the reconstituted PcVDAC open state (Figure 1) indicates the existence of two open states, O1 and O2, being selective to anions or cations, respectively. Our data indicates that low ion concentration (Figure 4A) and/or high concentration ratio across the membrane (Figure 2A) favor the O1=>O2 transition. The variation of the reversal potential with the transmembrane ion concentration ratio displays an extrema at the ratio value of 4. This is consistent with the data of Zambrowicz et al. who studied selectivity of the *Neurospora crassa* VDAC [47]. Assuming that the VDAC pore consists in two concentric regions with different electrostatic properties, these authors were able to reproduce the dependence of the selectivity on the concentration ratio. This assumption has been questioned [28] and our data suggests that the extremum is the consequence of the O1=>O2 transition. The inversion of the open state selectivity implies an excess of negatively charged residues without change in conductance.

### 3.2. VDAC Structure and Selectivity

Based on NMR spectroscopy and X-ray crystallography, the VDAC structure adopts a circular shape representing the O1 open state [21,22,23]. Though the circular shape was the only structure obtained using X-ray crystallography, twenty NMR structural conformers were found with circular or elliptic shape indicating that VDAC is highly flexible [22,48]. Molecular simulation studies performed on the twenty NMR structures of hVDAC1 revealed a wide range of conductance (0.21–0.67 nS in 0.1 MKCl) and anion selectivity but one of them displayed no selectivity (E_rev_ 0–58 mV with 1/0.1 KCl concentration ratio) [49]. In addition to these global topological changes, the selectivity (E_rev_) of the O1 state is affected by the solvent accessibility to charged residues in the pore and the reversible ionic interactions between charged residues [50], indicating that the anion selectivity of the O1 state also depends on the local protein conformation. Our single-channel conductance data (Figure 4) indicates that the PcVDAC displays a broad dispersion of the E_rev_ values. This was observed for both O1 and O2 open states. These results together indicate that there is no obvious correlation between PcVDAC conductance and its selectivity.

Computational studies on high-resolution structures have highlighted the molecular determinants of inorganic ions and ATP transport through the VDAC O1 state [46,49,50,51,52,53,54,55,56,57]. However, no selectivity inversion (O1=>O2) was observed. The experimental lifetime of both O1 and O2 states is greater than 1 s [38] while the molecular dynamics simulation are about 10^−9^ to 10^−5^ s long. Therefore, the occurrence of the selectivity inversion (O1=>O2 transition) in a molecular simulation is currently highly unlikely.

The selectivity inversion of PcVDAC does not require a change in conductance. Both theoretical and experimental investigations indicate that the selectivity of the PcVDAC O1 state is determined by the charge density distribution alongside the inner wall of the diffusion pore and the excess of positively charged residues [28]. We might thus hypothesize that the O1=>O2 transition arises from one (or more) local conformational change that would revert the net charge of the PcVDAC pore.

### 3.3. Effect of Stigmasterol

Compared to animals and fungi, plants have a greater molecular diversity of sterols among which campesterol, sitosterol and stigmasterol are the predominant phytosterols in plant membranes [58,59]. There is about 10 ± 5% of phytosterols in plant MOM [14]. Membrane lipids can actively influence membrane protein function. This regulation involves either short range interactions (specific) when some lipid molecules bind to proteins, or long range interactions (nonspecific) when the proteins experience the collective physical properties of the lipid bilayer (e.g., lateral transmembrane pressure, thickness, curvature, fluidity).

Numerous studies have highlighted the influence of sterol species on lipid-lipid interactions and their effect on the membrane organization and properties. Compared to other sterols, stigmasterol has a relatively low efficiency for ordering soy phospholipid acyl chains and consistently, it affects neither the water permeability of the soy lipid bilayer [60,61] nor the egg phosphatidylcholine lipid bilayer permeability to glycerol and erythritol [62]. Surprisingly, stigmasterol can slightly decrease the lipid ordering of DOPC/DPPC lipid bilayer [63]. We can thus reasonably assume that stigmasterol has a negligible effect on the collective physical properties of the lipid bilayer. It has previously been shown that stigmasterol affects reversibly both selectivity and gating of PcVDAC suggesting that a specific interaction might exist between stigmasterol and PcVDAC [20]. This assumption is consistent with the data obtained on mammalian VDAC showing that cholesterol co-purifies with bovine VDAC [64] and the identification of cholesterol binding sites in human VDAC1 based on NMR spectroscopy and molecular simulations [22,65].

We found that hydrogen/deuterium exchange kinetics increased in the presence of stigmasterol. Given that ATR-FTIR experiments focus on the amide protons only, it yields data proportional to the number of residues in the protein. PcVDAC comprises 275 amino acid residues, thus the 10% increase in hydrogen/deuterium exchange observed after 11 h in the presence of stigmasterol corresponds to about 30 additional deuterated amide groups or residues of amino acids. Although further investigations are required to elucidate the interaction between stigmasterol and PcVDAC at a molecular level, our data suggests that upon binding to VDAC, stigmasterol increases the accessibility of some amide groups to the solvent and/or local dynamics of PcVDAC.

The selectivity of VDAC was ascribed to Donnan equilibrium and depends on the charge density distribution alongside the inner wall of the diffusion pore, and the excess of positively charged residues [28,47]. The overall theoretical net charge of PcVDAC at neutral pH is +2 (29 positively charged residues (K and R) and 27 negatively charged residues (D and E)). The increase in anion selectivity observed in the presence of stigmasterol and a high ionic strength (1 M KCl in the Trans compartment) must be correlated to an increase in positively charged residues pointing toward the pore lumen. It has been shown that phosphatidylcholine head groups weakly interact with mouse VDAC1 acidic residues whereas phosphatidylethanolamine amine groups interact strongly with specific negatively charged residues [19]. A model structure built by comparative modeling suggests that there is about eight positively charged and six negatively charged residues located at the rim of the PcVDAC β-barrel [6]. We anticipate that stigmasterol binding might alter the interaction between the polar head of phospholipids and charged residues located on the rim of the β-barrel allowing for increase in the number of positively charged residues facing the channel entrance. This in turn causes an increase in the PcVDAC anion selectivity without significant change in the conductance.

The use of 1 M KCl concentration is a common practice in studies of VDAC functional properties by means of planar lipid bilayer electrophysiology. In plants, the effect of ion concentration has been studied on PcVDAC only and it has been shown that the gating is sensitive to the ionic strength of the experimental solution [19,20]. Here we show that the ion concentration also affects the selectivity inversion of the PcVDAC open state (the O1⬄O2 transition). As shown in Figure 2 and Figure 4, the occurrence of the O2 more cation selective open state decreases at concentration ratio larger than 4 at high KCl concentration in the presence of stigmasterol suggesting that it might stabilize PcVDAC in the O1 anionic open state.

The results obtained in 0.1 M KCl are more relevant to draw conclusions about the physiological significance of the selectivity inversion of the PcVDAC open state. It has been speculated that the voltage-independent O1=>O2 transition might regulate the metabolite permeability through MOM and that the cationic open state, O2, would be prevalent in the native membrane [38]. Our data does not support this statement. As shown in Figure 2, the occurrence of the O2 state is perceptible when KCl concentration ratio is higher than 4. The largest plausible KCl concentration ratio across MOM can be estimated using the Nernst equilibrium potential equation and the value of the MOM electric potential difference. The latter ranges between 30 and 40 mV [66,67], which corresponds to a KCl concentration ratio ranging between 3 and 4.5. In this range of concentration ratio, PcVDAC displays selectivity corresponding to the anionic open state. Thus, the anionic open state (O1) should be the prevalent PcVDAC state in MOM and the occurrence of the O2 state should be rare. In support of this conclusion, in their pioneer work, Pavlov et al. measured the selectivity in the presence of KCl concentration ratio of 5 and observed low frequency of occurrence of the cation-selective open state [38].

In conclusion, stigmasterol as well as the ionic strength and the magnitude of the ion concentration gradient affect the occurrence of the selectivity inversion of the reconstituted PcVDAC open state. Moreover, stigmasterol closely interacts with the reconstituted PcVDAC. Interestingly, the obtained data suggests that this selectivity inversion should not have a significant impact on the mitochondrial physiology in vivo.

## 4. Materials and Methods

### 4.1. VDAC Purification

*Phaseolus coccineus* seeds were soaked in tap water for 18 h and mitochondrial membranes were isolated from the cotyledons by differential centrifugation steps and further purified on 28% Percoll gradient as described previously [30]. Purification of the most abundant PcVDAC isoform (32 kDa, UniProt/Swiss-Prot accession number: Q4PKP6) was achieved using the chromatofocusing technique [25].

### 4.2. VDAC Reconstitution and Electrophysiology

The purified PcVDAC was reconstituted in planar lipid bilayers as described previously [68]. The soybean phospholipid extract was purchased from Avanti Polar Lipids, Inc. (Alabaster, AL, USA). It was dissolved in hexane to a final concentration of 2% (*w*/*v*). Planar lipid bilayers were formed by folding two lipid monolayers over a hole (100–130 µm in diameter) made in a 25 µm thick Teflon partition, which was treated with a solution of hexadecane/hexane (2.5%, *v*/*v*). Ag/AgCl electrodes connected in series with a salt bridge (1 M KCl in 1% agar) were used to connect the experimental chambers to the electronic equipment. The Trans compartment is defined as the one connected to the ground and the voltage was applied to the Cis compartment. For channel reconstitution into a planar lipid bilayer, proteins were added to the Cis compartment. KCl solutions were buffered with 10 mM HEPES at pH 7.5.

Current recordings were performed using BLM120 amplifier (BioLogic, Grenoble, France). Data were filtered at 300 Hz (5-poles linearized Tchebichev filter), digitized at 44.4 kHz with a DRA200 interface (BioLogic, Grenoble, France) and stored on CD for further processing using a homemade program written in the MATLAB 7.5 environment (MathWorks, Eindhoven, The Netherlands).

Selectivity experiments were performed on membranes doped with either a single- or multichannel as mentioned in the text. The reversal potential (zero-current potential) was set to zero in presence of identical KCl molality on both sides of the membrane. The Cis compartment was afterwards perfused at least three times its volume with a solution of different KCl concentration and the change in reversal potential (E_rev_) was directly read on the amplifier. The direct measurement of the Erev was preferred over the I-V curve because in some condition, the selectivity of the open state switched randomly between anion and cation selectivity and the Erev must be assessed instantaneously in the course of the experiment. We also checked that this Erev obtained in this way was akin to that assessed using a current-voltage relationship (Figure S3: Selectivity measurement). The values of E_rev_ were corrected for the liquid junction potential at salt bridges.

The single channel conductance was calculated from the current amplitude flowing through the channel in response to 10 mV voltage pulse using Ohm law.

### 4.3. Kinetics of the Hydrogen/Deuterium Exchange

Proteoliposomes were obtained by mixing multilammellar liposomes (0.6 mg lipids) with PcVDAC (0.25 mg) solubilized in 4% octyl-POE. The mixture was extensively dialyzed against 1 mM Hepes (pH 7.2) and proteoliposomes were separated in sucrose gradient (35−5%) by ultracentrifugation (40,000 g for 1 h) and washed in 1 mM Hepes (pH 7.2).

The hydrogen/deuterium exchange kinetics monitored by Attenuated Total Reflection-Fourier transform Infra-Red (ATR-FTIR) spectroscopy were carried out as described previously [25,69]. About 20 to 30 μL (15 to 30 μg) of reconstituted proteins were spread on one side of the ATR crystal. The solvent was slowly evaporated under a continuous nitrogen gas stream. A computer program controlled the kinetic measurements. Prior to each experiment, 10 spectra were recorded to verify the stability and the reproducibility of the system. At time 0, the sample was flushed with D_2_O-saturated nitrogen gas at a flow rate of 4 L/min. For each spectrum, 24 scans were accumulated at a resolution of 2 cm^−1^. Background and water vapor spectra were subtracted from the kinetic spectra as reported. The areas of the amide I and amide II bands were calculated by integration of the spectra between 1700 and 1600 cm^−1^ and 1590 and 1505 cm^−1^, respectively. The area of the amide II band decreases as deuteration of the protein proceeds. To take into account variations of the overall spectral intensities related to film swelling upon hydration in the first minutes of the measurement, the amide II/amide I ratio was determined for each spectrum. Thus, the hydrogen/deuterium exchange was monitored as the evolution of the ratio of amide II/amide I areas, expressed as percent of non-exchanged amide protons. The amide II/amide I ratios of the non-deuterated spectra were taken as 100%, whereas the 0% value corresponds to a zero absorption in the amide II region.

### 4.4. Statistics

Statistical analysis and linear regression were performed with the MATLAB 7.5 environment. Experimental data are shown as the mean (of a given number of replicates) ± standard error of the mean (number of replicates). The statistical significance between different means was estimated using one-way analysis of variance (one-way ANOVA) and was used to test for differences among several groups of data. Multiple pairwise comparison test was used to identify a statistically different pair of groups with a confidence of 0.05 (i.e., α-probabilities (*p*) < 0.05).

## Data Availability

The data presented in this study are available on request from the corresponding author.

## Supplementary Materials

The following are available online at https://www.mdpi.com/1422-0067/22/6/3034/s1.

## Abbreviations

VDAC	Voltage-Dependent Anion Channel
MOM	Mitochondrial Outer Membrane
ATR-FTIR	Attenuated Total Reflection-Fourier transform Infra-Red
DOPC	1,2-Dioleoyl-sn-Glycero-3-Phosphocholine
DOPE	1,2-Dioleoyl-sn-Glycero-3-Phosphoethanolamine
DPPC	1,2-Dipalmitoyl-sn-Glycero-3-Phosphocholine
