# Supplementary material for: The Open State Selectivity of the Bean Seed VDAC Depends on Stigmasterol and Ion Concentration
Source: Int J Mol Sci. 2021 Mar 16;22(6):3034. doi: 10.3390/ijms22063034 (PMC8002290; doi:10.3390/ijms22063034)
Supplement: Supplementary file 1 [file ijms-22-03034-s001.zip › Figure_S2_Channel_conductance.pdf]

Figure S2: Channel conductance

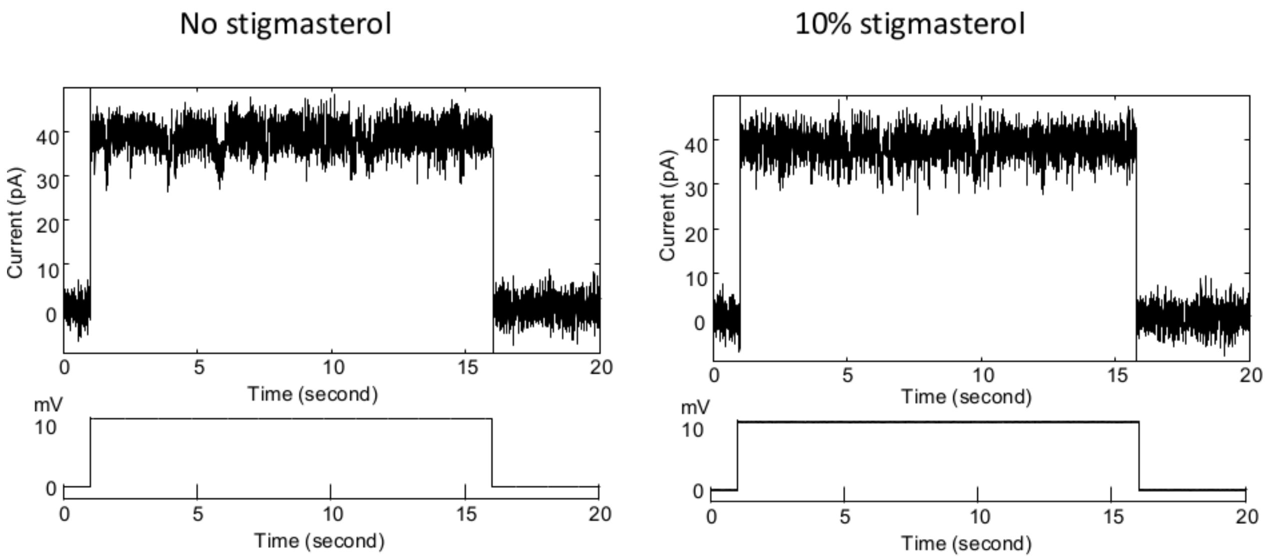

Effect of stigmasterol on the PcVDAC conductance. To assess the conductance (G), the current amplitude (I) flowing through the channel in response to 10 mV voltage pulse (V). The conductance was calculated using Ohm law (G = I/V).

The figure displays two independent single-channel experiments corresponding to a single PcVDAC reconstituted in a planar lipid bilayer. The membrane was either free of stigmasterol (left) or doped with 10% stigmasterol (right). Each compartment was filled with 1M KCl buffered at pH 7.5 with 10mM HEPES-KOH.
