# Supplementary material for: The Open State Selectivity of the Bean Seed VDAC Depends on Stigmasterol and Ion Concentration
Source: Int J Mol Sci. 2021 Mar 16;22(6):3034. doi: 10.3390/ijms22063034 (PMC8002290; doi:10.3390/ijms22063034)
Supplement: Supplementary file 1 [file ijms-22-03034-s001.zip › _Figure_S1_Multiple_pairwise_comparison.pdf]

Figure S1 : Multiple pairwise comparison

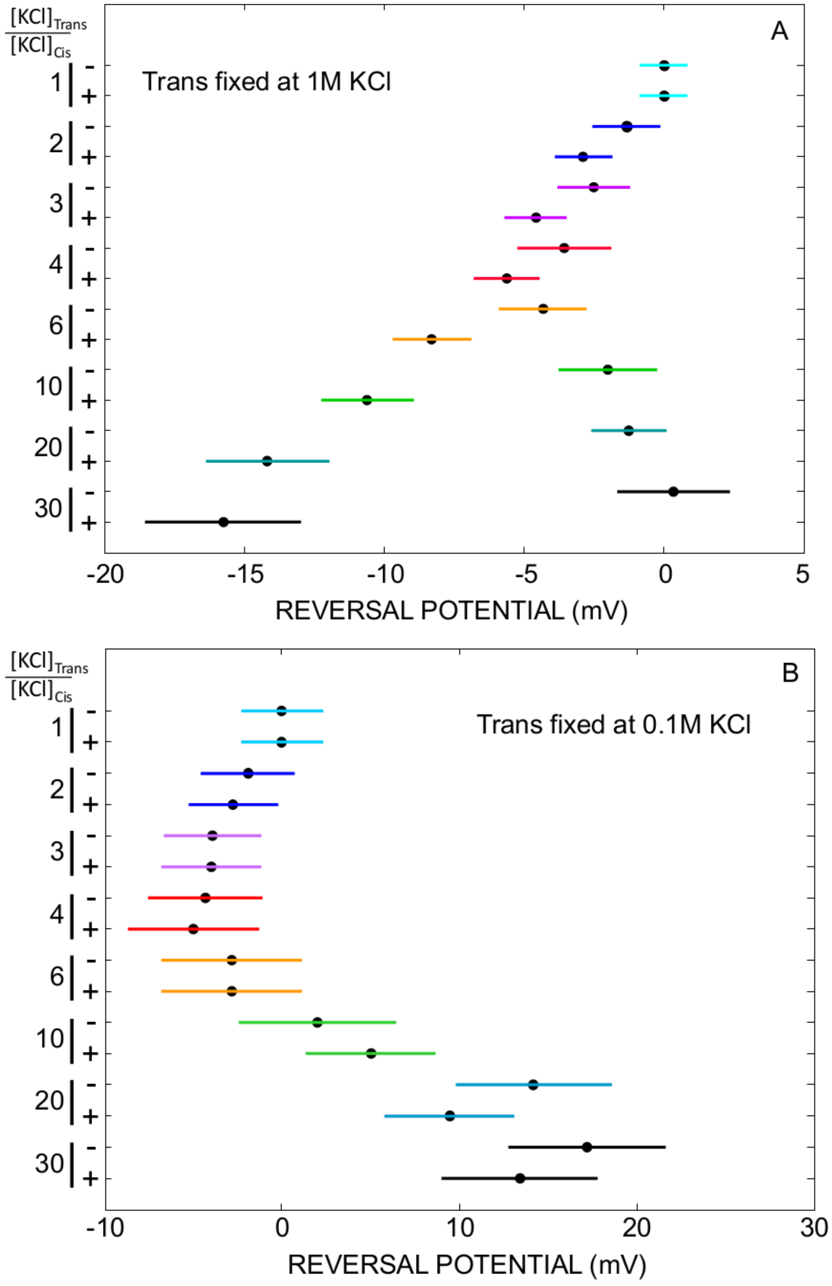

We performed pairwise comparison to confirm that there are pairs of means that are significantly different in figure 2C and 2D. For each group, the horizontal line represents the 95% confidence ($(p) < 0.05$) interval for the true mean (black circle). Panel A: The data indicates that at high ionic strength (fixed Trans concentration 1M) there is a statistically significant effect of stigmasterol on the PcVDAC selectivity for KCl concentration ratio higher than 4. Panel B: The data indicates that at low ionic strength (fixed Trans concentration 0.1M) there is no statistically significant effect of stigmasterol on the PcVDAC selectivity for all KCl concentration ratio.
