# Supplementary material for: The Open State Selectivity of the Bean Seed VDAC Depends on Stigmasterol and Ion Concentration
Source: Int J Mol Sci. 2021 Mar 16;22(6):3034. doi: 10.3390/ijms22063034 (PMC8002290; doi:10.3390/ijms22063034)
Supplement: Supplementary file 1 [file ijms-22-03034-s001.zip › _Figure_S3_Selectivity_measurement.pdf]

Figure S3: Selectivity measurement.

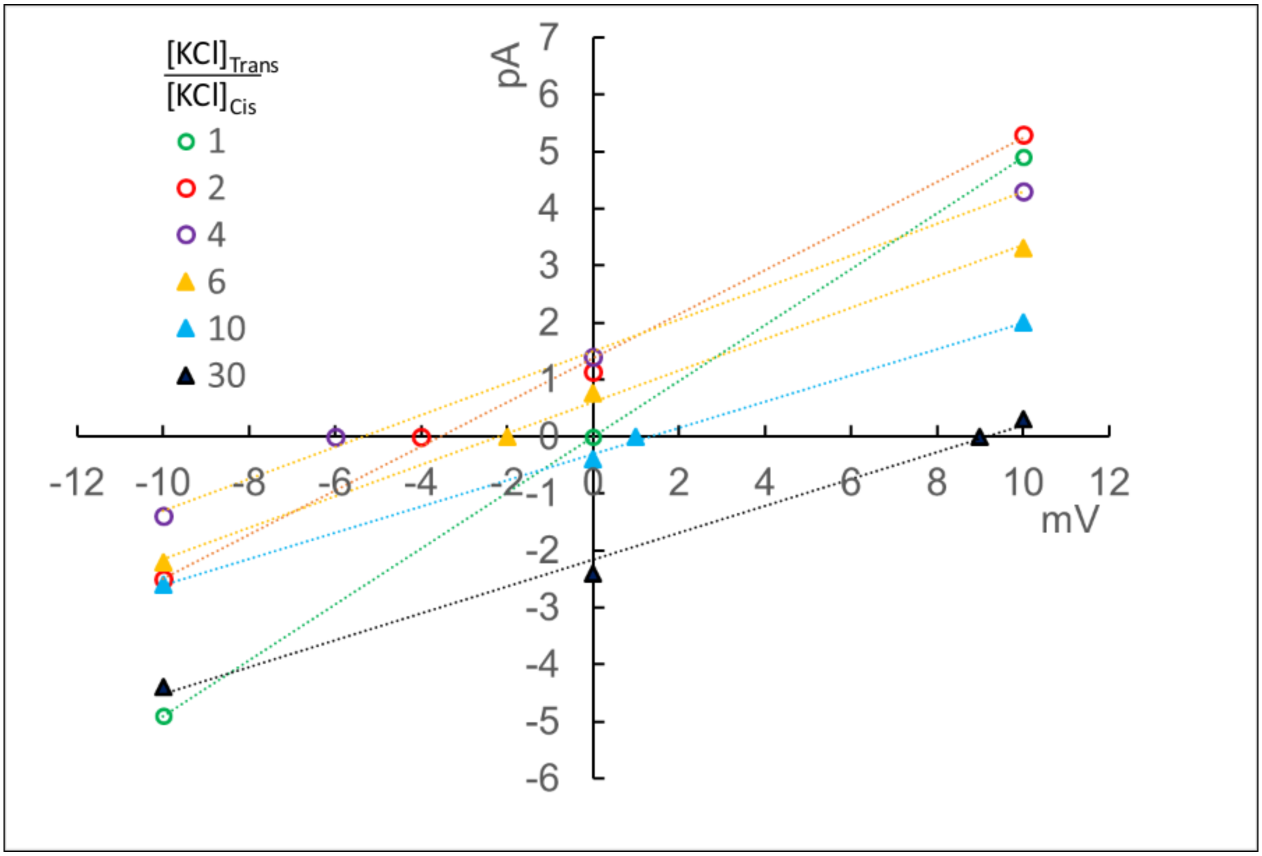

Change in the reversal potential with KCl concentration ratio (Trans/Cis). The ion concentration in the Trans compartment was fixed at 0.1M. To generate a given concentration ratio (2, 4, 6, 10 and 30) between the Trans and Cis compartments, the latter was perfused with low concentration KCl solutions. The solutions were buffered at pH 7.5 with 10mM HEPES-KOH. Four points were used to draw a current-voltage at each concentration ratio. The Erev (V(I=0)) and I(V=0) were directly read on the amplifier. Two additional points were obtained at +10mV and – 10mV. A linear least square regression was used to fit the data with a goodness of fit R^2>0.99 for all fits. Open circle symbols were used for the lowest gradient showing the canonic anionic selectivity (concentration ratio = 2 and 4) while closed triangle were used for data indicating a decrease in anionic selectivity (concentration ratio = 6) or a cationic selectivity (concentration ratio = 10 and 30). The Erev values are found on the abscissa. This figure shows that there is a good correlation between the Erev read directly on the amplifier and that extrapolated from the current-voltage curve.
